# Microneedle Technologies for Drug Delivery: Innovations, Applications, and Commercial Challenges

**DOI:** 10.3390/mi17010102

**Published:** 2026-01-13

**Authors:** Kranthi Gattu, Deepika Godugu, Harsha Jain, Krishna Jadhav, Hyunah Cho, Satish Rojekar

**Affiliations:** 1 Industrial Pharmacy, Department of Pharmaceutical Sciences, College of Pharmacy and Health Sciences, St. John’s University, Queens, NY 11439, USA; kranthi.gattu23@my.stjohns.edu; 2 Department of Pharmacy, University College of Technology Sciences, Osmania University, Hyderabad 500007, Telangana, India; drdeepikagodugu@gmail.com; 3 College of Pharmacy, University of Iowa, Iowa City, IA 52242, USA; harsha-jain@uiowa.edu; 4 Institute for Bioengineering of Catalonia (IBEC), Baldiri Reixac 10-12, 08028 Barcelona, Spain; krishnajadhavphd@gmail.com; 5 College of Pharmacy and Health Sciences, Fairleigh Dickinson University, Madison, NJ 07932, USA; hyunahc@fdu.edu; 6 Department of Pharmacological Sciences, Institute for Translational Medicine and Pharmacology, Icahn School of Medicine, Mount Sinai, NY 10029, USA

**Keywords:** microneedle, transdermal delivery, drug delivery, biomaterials, vaccines, 3D printing

## Abstract

Microneedle (MN) technologies have emerged as a groundbreaking platform for transdermal and intradermal drug delivery, offering a minimally invasive alternative to oral and parenteral routes. Unlike passive transdermal systems, MNs allow the permeation of hydrophilic macromolecules, such as peptides, proteins, and vaccines, by penetrating the stratum corneum barrier without causing pain or tissue damage, unlike hypodermic needles. Recent advances in materials science, microfabrication, and biomedical engineering have enabled the development of various MN types, including solid, coated, dissolving, hollow, hydrogel-forming, and hybrid designs. Each type has unique mechanisms, fabrication techniques, and pharmacokinetic profiles, providing customized solutions for a range of therapeutic applications. The integration of 3D printing technologies and stimulus-responsive polymers into MN systems has enabled patches that combine drug delivery with real-time physiological sensing. Over the years, MN applications have grown beyond vaccines to include the delivery of insulin, anticancer agents, contraceptives, and various cosmeceutical ingredients, highlighting the versatility of this platform. Despite this progress, broader clinical and commercial adoption is still limited by issues such as scalable and reliable manufacturing, patient acceptance, and meeting regulatory expectations. Overcoming these barriers will require coordinated efforts across engineering, clinical research, and regulatory science. This review thoroughly summarizes MN technologies, beginning with their classification and drug-delivery mechanisms, and then explores innovations, therapeutic uses, and translational challenges. It concludes with a critical analysis of clinical case studies and a future outlook for global healthcare. By comparing technological progress with regulatory and commercial hurdles, this article highlights the opportunities and limitations of MN systems as a next-generation drug-delivery platform.

## 1. Introduction

Transdermal drug delivery has evolved from ancient topical remedies to sophisticated medical devices designed for controlled and efficient administration of therapeutics through the skin [1]. Over the past few decades, advances in microfabrication and material science have driven the emergence of microneedle (MN) technologies, representing a transformative leap that overcomes the limitations of oral and conventional parenteral drug delivery methods [2]. This technology enables minimally invasive, pain-free delivery of a wide range of drugs and biologics, improving patient compliance and therapeutic outcomes [3].

### 1.1. History of MN

MN technology has advanced considerably since its inception in the 1970s, with key innovations occurring in the 1990s, when microfabricated silicon MNs were introduced as less-invasive instruments for transdermal drug delivery. This period initiated advanced manufacturing techniques utilizing materials including silicon, metals, and polymers [4]. The 2000s witnessed diversification into numerous MN forms, providing distinct benefits for the delivery of vaccines, small compounds, and biomacromolecules. Clinical translation advanced through trials that exhibited less pain, enhanced patient adherence, and expanded therapeutic possibilities, encompassing cosmetic uses [5,6].

**Figure 1:**
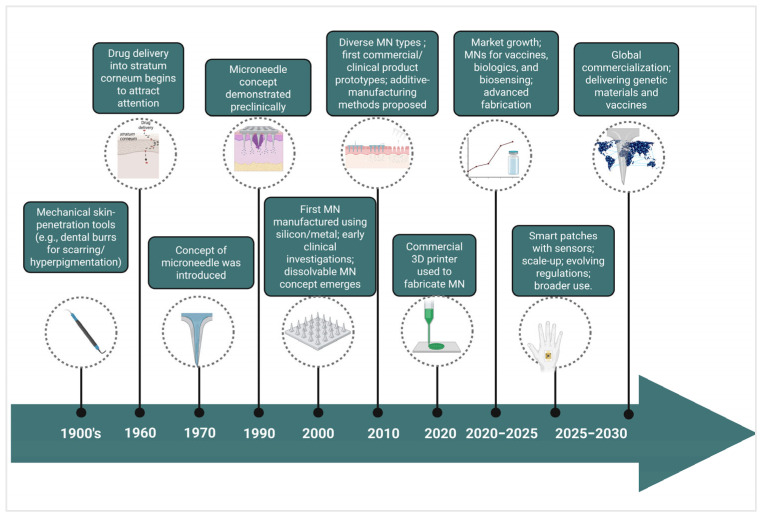
Timeline showing the evolution and projected future of MN technology from research to global commercialization (created using Biorender, Godugu, D. (2025) https://BioRender.com/0x4v3gz, accessed on 2 November 2025).

Over the past decade, significant progress has been achieved through the integration of innovative materials and manufacturing methods, such as 3D printing, to facilitate scalable, precise production of MNs. Concurrently, research efforts have expanded toward multifunctional MN platforms incorporating sensing elements, stimuli-responsive materials, and closed-loop control capabilities. However, such smart MN systems largely remain at the preclinical or early translational stage [7]. The period from 2020 to 2030 characterizes the transformation of MN technology from a laboratory concept into a clinically viable platform, as the field addresses remaining hurdles in drug loading capacity, scale-up, and regulatory compliance. The continuous developments underscore the evolution of MNs from experimental apparatus to multifunctional instruments pivotal to next-generation medicines and diagnostics [8] (Figure 1).

### 1.2. Structure and Design Principles

**Figure 2:**
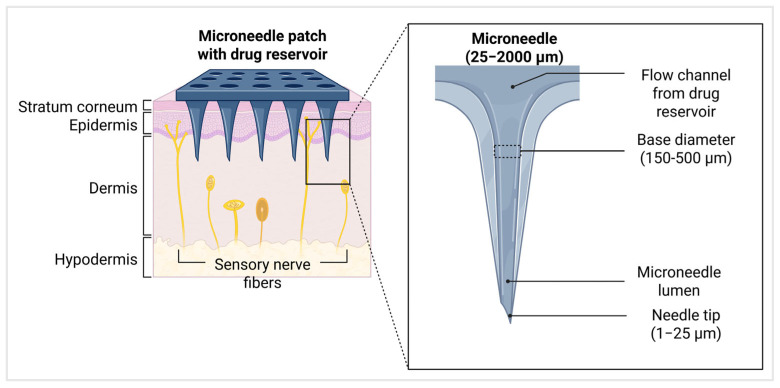
MN patch penetrating skin layers for transdermal drug delivery. Inset shows microneedle structure with lumen and delivery channel (created using Biorender, Godugu, D. (2025) https://BioRender.com/r3qefwt, accessed on 7 November 2025).

MNs are arrays of microscale needles typically ranging in length from 25 micrometers (μm) to 2000 μm (2 mm), which corresponds approximately to the thickness of the human epidermis, including the stratum corneum [4]. The needle tip radius is designed to be sharp, ranging from 1 μm to 25 μm, to ensure efficient penetration through the skin barrier with minimal pain. The base diameter of individual MNs typically ranges from 150 μm to 500 μm [9]. These are arranged in arrays commonly sized around 8 mm by 8 mm, with spacing (pitch) between the needles approximately 500 μm (Figure 2) [10]. The needle height is carefully engineered to breach the outer skin layers without reaching pain receptors or blood vessels in the dermis, thereby providing a minimally invasive and painless drug delivery experience. These dimensions are crucial for achieving a balance between effective skin penetration and patient comfort and safety [11].

This review summarizes recent developments across different MN platforms, including their classification, modes of delivery, emerging technologies, therapeutic uses, commercial hurdles, and key illustrative examples. The goal is to offer a clear and balanced assessment of current progress and remaining challenges, providing readers with a practical guide to understanding and advancing work in this rapidly evolving area of drug-delivery research.

## 2. Classification of Microneedles

MNs can generally be grouped into five types: solid, coated, dissolving, hollow, and hydrogel-forming MNs, along with other hybrid types of MNs that have recently been developed based on their structure, materials, and methods of drug administration (Table 1) [12]. Different MNs have different fabrication methods, giving them specific mechanical properties and therapies that fit their properties [13,14]. Each type has its own optimizations and particular characteristics that optimize transdermal drug delivery, such as strengthening the drug’s loading, improving its release kinetics, and maintaining the patient’s overall safety, in addition to overcoming the barrier of the stratum corneum’s impermeability [15].

### 2.1. Solid Microneedles

Solid MNs are the simplest type of MNs. These MNs can be fabricated from a range of biocompatible materials, including silicon, metals such as stainless steel and titanium, and biodegradable polymers such as polylactic acid (PLA) [16]. Their operation involves the “poke-and-patch” mechanism, in which the MN array pretreats the skin with a drug patch, creating microchannels (50 to 100 μm in depth). The needles can be made as long as 100 to 900 μm to ensure the dermal–epidermal border is not penetrated. These MNs are preferred for skin pre-treatment during MN infiltration due to their mechanical stiffness and >90% reusability (costing less than 10 cents per unit at large scale) [15]. Fabrication methods include wet or dry etching of silicon, laser cutting of metals, and micro molding of polymers, with needle lengths of 100–900 μm to penetrate the viable epidermis without dermal vasculature [17]. Advantages include mechanical robustness, reusability, and inexpensive production, making them ideal for pretreatment applications in combination with iontophoresis or electroporation [1]. However, limitations include rapid channel closure (15–30 min reflecting skin elasticity), resulting in variable drug permeation rates and possible incomplete delivery in dynamic skin environments. Recent work has introduced bioactive coatings that actively maintain open pores, improving the overall performance of these systems [15].

### 2.2. Coated Microneedles

Coated MNs are an advanced design based on solid MNs, where drug formulations are layered onto the needle surface using techniques such as dip coating, spray coating, or inkjet printing. These designs employ biocompatible core materials like silicon, stainless steel, or polymers (e.g., PLA), with the active pharmaceutical agents adhering as uniform thin films [18]. Upon skin insertion, rapid dissolution or detachment of the coating delivers precise doses into microchannels approximately 50–150 microns deep, bypassing the stratum corneum and enabling efficient drug absorption [19]. Coating thickness and uniformity are critical and are often controlled by process parameters such as solution viscosity and withdrawal speed. Their primary benefits include immediate drug release, suitability for delivering low-dosage biotherapeutics, and versatility for vaccines, peptides, or hormones [20]. However, limitations involve challenges in achieving consistent coating coverage, dose loading, and stability during storage and transport. Recent innovations involve mucoadhesive, stimuli-responsive, and multilayer bioactive coatings that enhance skin permeation and therapeutic efficacy [21].

### 2.3. Dissolving Microneedles

Dissolving MNs contain the drug in a water-soluble matrix, which is often made of biodegradable polymers (such as hyaluronic acid, polyvinylpyrrolidone, polyvinyl alcohol) or carbohydrates (such as sucrose, maltose) [22]. Once inserted into the skin, the MN dissolves in the interstitial fluid (ISF) within 5–30 min, enabling controlled drug release while avoiding the generation of sharps waste. Fabrication techniques using micro molding or droplet-born air blowing provide arrays ranging from 100–600 μm and drug loading up to 1 mg/patch [23]. These are especially beneficial for biologics such as insulin or vaccines, because they provide accurate dosing and better patient compliance through painless and self-administration [24]. Limitations include mechanical fragility (fracture risk during insertion into dry skin) and the dissolution rate, which is affected by skin hydration or pH, potentially resulting in incomplete dissolution (efficiency of 70–95%). Recent formulations include ingredients such as chondroitin sulfate to provide greater strength [25].

### 2.4. Hollow Microneedles

Hollow MNs are similar to mini-hypodermic needles, with lumens connecting to the center to deliver liquid formulations into the dermis. They provide a chance for bolus or the continuous delivery of larger drug volumes compared to other MNs, but require more complex production and insertion force [20,26].

### 2.5. Hydrogel-Forming Microneedles

Hydrogel-forming MNs use crosslinked, swellable polymers (e.g., poly(methyl vinyl ether-co-maleic anhydride) and polyethylene glycol diacrylate) that absorb ISF upon insertion, expanding 200–500% in volume to form drug-permeable conduits from an attached reservoir [27,28]. Unlike dissolving types, the matrix is not dissolved for removal after use, providing sustained release for hours to days through diffusion. Fabrication processes include casting or photopolymerization processes, often with integrated backings [13]. Benefits include zero-order kinetics for chronic therapies and minimal residue, but limitations include a slower onset (swelling time) and a bulkier reservoir, which may affect wearability. pH or enzyme-sensitive hydrogels provide an enhanced method of control [29].

### 2.6. Hybrid and Next-Generation Microneedles

Hybrid MNs incorporate components from many different classes, such as dissolving tips on hollow shafts or coated hydrogels, to combine benefits such as high loading with controlled infusion [30]. Next-generation design considers stimuli-responsive materials (e.g., thermos or glucose-sensitive polymers) [31]; nanoparticles (NPs) for targeted delivery [32]; bio-inspired structures (e.g., mosquito-like barbs) [33]; and additive manufacturing for personalization [34]. These provide versatility for theragnostics but are challenged in terms of scalability and regulation [8].

**Table 1:** Classification of MNs: mechanism, fabrication, advantages, and limitations.

Type	Fabrication Materials/Methods	Mechanism	Advantages	Limitations	Reference
Solid	Silicon, metals, polymers, etching, molding	Creates microchannels for passive diffusion	Simple design, low cost	Poor control of dosing	[12]
Coated	Dip-coating, spray-coating, and inkjet printing	Drug layered on the surface, dissolves upon insertion	Rapid release, suited for vaccines	Limited drug load	[18]
Dissolving	Polymers (polyvinylpyrrolidone, hyaluronic acid) via micro molding	The biodegradable matrix dissolves in the skin, releasing the drug	No waste, suitable for biologics	Fragility, limited penetration	[35]
Hollow	Silicon, glass, stainless steel; laser micromachining	Drug infused through the central lumen	Larger volumes, controlled infusion	Complex design, higher cost	[36]
Hydrogel-forming	Crosslinked polymers (PEG, PHEMA)	Swellable polymers form drug-permeable conduits	Sustained release, reusable reservoir	Removal required, slower onset	[37,38]
Hybrid/Next-gen	Composite polymers, 3D printing, NPs	Combines multiple features; smart materials	High versatility, personalized therapy	Still experimental, scalability issues	[39]

## 3. Mechanisms of Drug Delivery via Microneedles

MNs enhance drug permeation across the skin by physically or chemically modulating the stratum corneum, the primary barrier to transdermal delivery. The mechanism of delivery depends on the MN type, formulation, and physicochemical properties of the drug [40,41].

### 3.1. Passive Diffusion via Solid Microneedles

**Figure 3:**
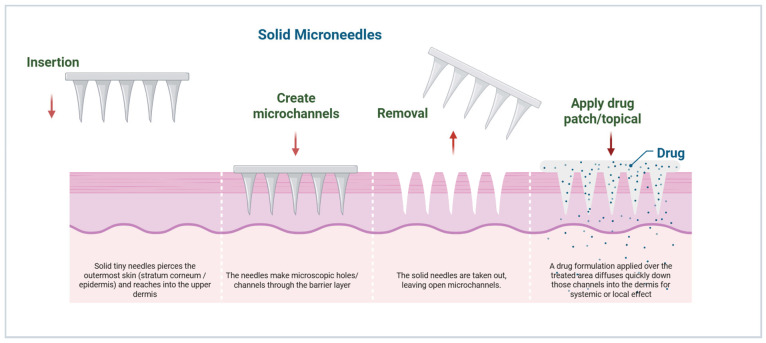
Schematic representation of solid MNs (poke-and-patch) and their mechanisms of drug delivery through the skin (created using Biorender, Godugu, D. (2025) https://BioRender.com/y50mebj, accessed on 7 November 2025).

Solid MNs work primarily by forming microchannels through the stratum corneum, facilitating the subsequent diffusion of topically applied drugs. The “poke-and-patch” approach relies on passive diffusion gradients, hydrophilicity, and hydration [15]. The formation of microchannels is rapid and reversible; microchannels usually close within hours due to the skin’s elasticity and its mechanisms for repairing damaged skin. Studies have demonstrated enhanced delivery of small molecules, peptides, and vaccines using solid MN-mediated microchannels, with permeability enhancements ranging from 10 to 1000-fold compared to intact skin [42] (Figure 3).

### 3.2. Coating Dissolution Kinetics in Coated Microneedles

**Figure 4:**
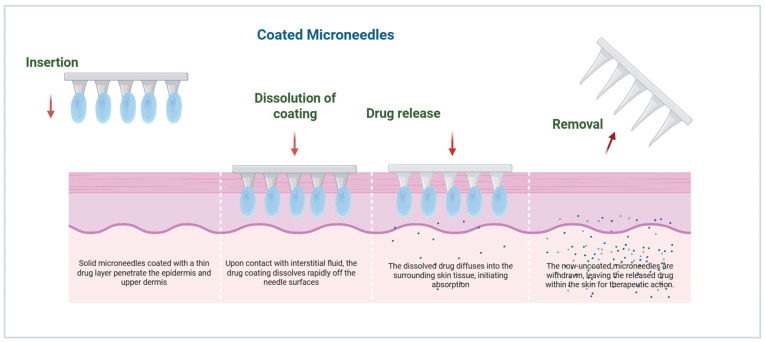
Illustrative mechanism of drug diffusion and release from coated MNs into the dermal layer (created using Biorender, Godugu, D. (2025) https://BioRender.com/mbtqmrm, accessed on 7 November 2025).

Coated MNs are used to deliver drugs via the dissolution of a thin API layer coated onto the surface of the needle [43]. Drug release occurs minutes after insertion and is controlled by coating thickness, polymer excipient composition, and fluid dynamics in the skin’s interstitial space [42]. This rapid release is suitable for vaccines and potent biologics, for which precise dosing and rapid onset are essential. The uniformity of coating and stability of adhesion during insertion are of great importance for reproducible delivery (Figure 4) [43].

### 3.3. Biodegradable Matrix Dissolution in Dissolving Microneedles

**Figure 5:**
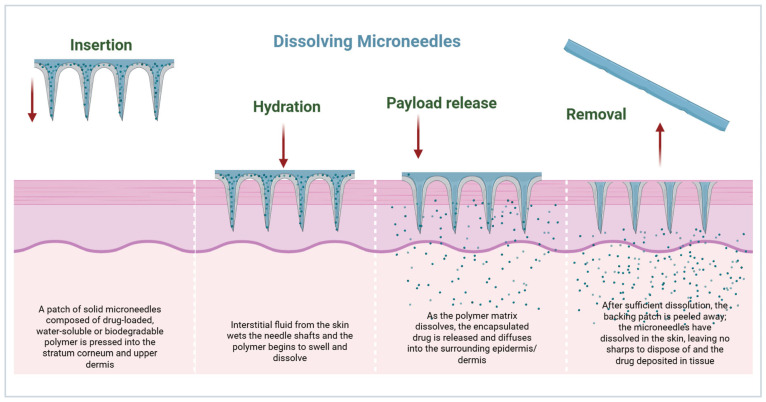
Representation of the insertion, hydration, and drug release mechanisms of dissolving MNs for controlled drug delivery through the skin (created using Biorender, Godugu, D. (2025) https://BioRender.com/j1hiys8, accessed on 7 November 2025).

Dissolving MNs encapsulate the API in a biodegradable polymer matrix (e.g., polyvinylpyrrolidone, hyaluronic acid, carboxymethyl cellulose) [44]. Upon insertion, ISF diffuses into the matrix, dissolving and releasing the encapsulated drug. The polymer molecular weight, degree of crosslinking, and needle geometry can manipulate release kinetics [45]. Dissolving MNs leave no residual sharps and decrease biohazardous waste, and can stabilize thermolabile biologics in solid state formulations [46]. Clinical studies have demonstrated their efficacy for insulin and influenza vaccines, highlighting patient-friendly administration and improved compliance (Figure 5) [47].

### 3.4. Infusion Through Hollow Microneedles

**Figure 6:**
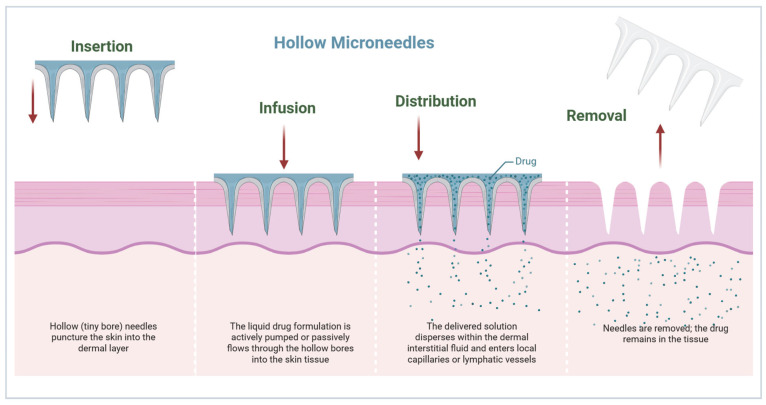
Diagram depicting the structure and drug distribution process of hollow MNs across the skin barrier (created using Biorender, Godugu, D. (2025) https://BioRender.com/ksv0rll, accessed on 7 November 2025).

Hollow MNs contain a central lumen that allows for direct infusion of liquid formulations into the dermis. Drug delivery can be given as a bolus, continuous infusion, or pulsatile dose with precise control of the pharmacokinetics [36]. The infusion rate is affected by the needle diameter, the lumen length, the depth of insertion, and skin backpressure. Hollow MNs can be used for a larger volume and viscous formulations suitable for biologics, monoclonal antibodies, and chemotherapeutics (Figure 6) [48].

### 3.5. Swelling and Sustained Release via Hydrogel-Forming Microneedles

**Figure 7:**
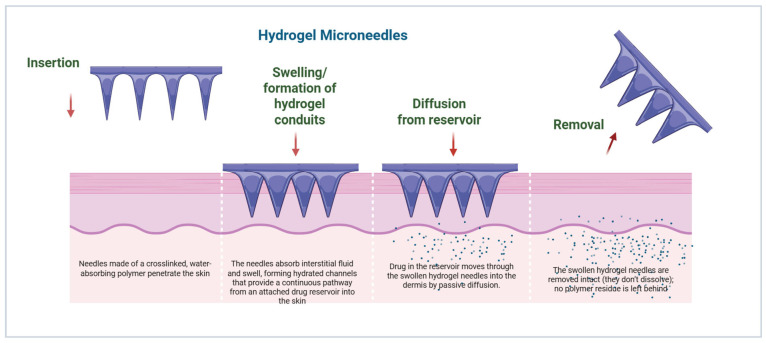
Schematic illustration of hydrogel-forming MNs and their transdermal drug delivery mechanism (created using Biorender, Godugu, D. (2025) https://BioRender.com/hqv7bh1, accessed on 7 November 2025).

Hydrogel-forming MNs consist of crosslinked swellable polymers (e.g., PEG, polyHEMA) that absorb ISF to create conduits for the diffusion of drug species from an attached reservoir [49]. Sustained release can occur in hours to days, depending on the polymer composition and degree of crosslinking [13]. Unlike dissolving MNs, the hydrogel matrix is not dissolved after therapy, which helps to reduce the deposition of residual polymer in the skin [50]. These hydrogel MNs have demonstrated potential for sustained insulin delivery, vaccines, and small molecule drug delivery, with enhanced pharmacokinetic profiles and reduced frequency of dosing (Figure 7) [51,52].

### 3.6. Hybrid and Stimuli-Responsive Mechanisms

Next-generation MN platforms increasingly integrate multiple delivery mechanisms within a single construct, combining, for example, dissolving tips with mechanically robust solid bases or incorporating stimuli-responsive polymers that enable controlled drug release in response to physiological or external triggers such as temperature, pH, or enzymatic activity [53]. To further enhance targeting and release kinetics, nanocarriers, including NPs, liposomes, and polymeric micelles, may be embedded within the MN matrix, allowing for spatiotemporal modulation of therapeutic payloads [8]. Such hybrid designs enable sophisticated delivery profiles, including pulsatile, sustained, or on-demand release, and can be coupled with wearable electronic systems to provide real-time physiological feedback and closed-loop drug administration [18,44].

## 4. Innovations in Microneedle Technologies

**Figure 8:**
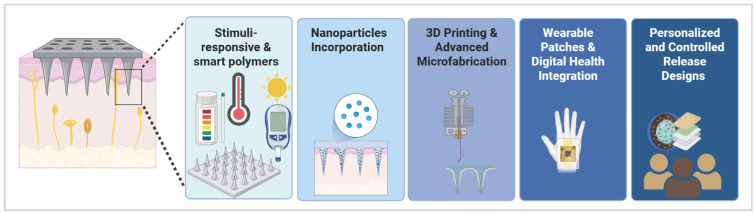
Schematic illustration of advanced MN technologies for transdermal delivery, highlighting smart polymers, NPs, 3D printing, wearable integration, and personalized release features (created in Biorender, Godugu, D. (2025) https://BioRender.com/u2xrtzu, accessed on 27 October 2025).

Technological advances in materials sciences, microfabrication, nanotechnology, and digital health are propelling MN systems beyond traditional transdermal drug delivery applications, moving them towards innovative, more precise, and more patient-centric solutions (Table 2 and Figure 8) [54]. Recent developments focus on designs with multifunctionality from diagnostics, targeted therapy, and remote monitoring, as well as issues of bioavailability, patient adherence, and scalability [8]. Innovations in laser-ablation molds for the fabrication of polymer MNs and circularly polarized light optical vortices for metal microstructures provide an enhancement of fabrication precision and mechanical properties [55]. These advancements are opening up the use of MN in wound healing, metabolic disorders, nucleic acid therapeutics, and even intraocular delivery, with projections for this technology in the market showing their use in next-generation pharmaceutics [56].

### 4.1. Stimuli-Responsive and Smart Polymers

Incorporating stimuli-responsive polymers into MNs enables dynamic, on-demand drug release controlled by environmental stimuli, representing a significant advancement in precision drug delivery [57]. These “smart” polymers respond to diverse stimuli, including pH, temperature, glucose, light, electrical, or magnetic fields, thereby modulating drug release kinetics for tailored therapeutic effects [58]. For instance, pH-responsive polymers such as poly(acrylic acid) facilitate targeted drug release in the acidic tumor microenvironment, enhancing chemotherapy efficacy while minimizing systemic toxicity [59]. Glucose-responsive insulin MNs using phenylboronic acid-based carriers exhibit swelling or shrinking behaviors in response to blood glucose levels. These strategies enable closed-loop insulin delivery systems that effectively mimic pancreatic function for diabetes management [60,61]. Additionally, Tong et al. explored a dual-responsive insulin delivery platform that integrates glucose- and H_2_O_2_-sensitive polymeric vesicles with transdermal MN arrays, enabling regulated insulin release in response to hyperglycemic conditions [62].

Polymeric MNs have proven effective for biofilm eradication in diseases ranging from cancer to diabetes [63]. Zhang et al. engineered core–shell MNs designed to sequentially modulate the wound microenvironment. Following laser-induced biofilm ablation, ROS-triggered shell degradation exposes an anti-inflammatory core and releases verteporfin to inhibit Engrailed-1, revealing scarless repair across both murine and lapine models [64]. Additionally, the emergence of photo-responsive, electro-responsive, and ultrasound-responsive MN systems has imparted distinct advantages for applications demanding precise spatiotemporal control over drug activation and release [65]. Together, these advances establish stimuli-responsive MNs as a cutting-edge platform for personalized and responsive drug delivery systems in clinical and translational medicine.

### 4.2. Nanoparticle Incorporation and Multifunctional Microneedles

Incorporating NPs, liposomes, nanosuspension, micelles, or dendrimers within MN matrices not only improves the stability of encapsulated drugs but also facilitates targeted delivery to specific cells or tissues. Additionally, this approach allows for the integration of multiple therapeutic or diagnostic functions, broadening the scope and versatility of MN-based delivery systems [66]. Moreover, the incorporation of NPs within MN systems extends the potential for personalized co-loading of multiple therapeutic agents, thereby enabling localized drug distribution at disease sites and improving targeting efficiency [67]. In cancer therapy, MN platforms incorporating gold or silica NPs have been explored for the localized combination of photothermal ablation and chemotherapy. Upon near-infrared irradiation, these NP-MN systems enable efficient heat generation within tumor tissues, thereby enhancing chemotherapeutic efficacy and resulting in pronounced tumor growth suppression in murine models [68]. The integration of pH-responsive NPs into MN matrices represents an additional strategy for cancer therapy. For instance, hyaluronic acid-based MNs incorporating pH-sensitive dextran NPs have been developed for the localized delivery of anti-programmed death-1 (aPD-1) antibodies [69].

Niu et al. designed an MN-based vaccination strategy aimed at augmenting immune responses by co-encapsulating the model antigen ovalbumin (OVA) with Toll-like receptor (TLR) agonists, namely imiquimod and monophosphoryl lipid A, within poly(D,L-lactide-co-ethyl lactone) (PLGA) NPs [70]. Similarly, a dissolving MN-delivered photothermal nano-vaccine integrating polyserotonin-based NPs and a Mn^2+^-responsive metal–organic framework achieved robust activation of dendritic cells, enhanced intratumoral T-cell infiltration, and significant suppression of both primary and distant melanoma tumors in murine models [71]. Multifunctional MNs incorporate biosensing, including electrochemical detection of biomarkers in ISF using NP-based sensing for real-time monitoring during the drug release [72,73]. Recent innovations include stimuli-responsive NPs for gene therapy, where magnetic NPs provide guided delivery under external fields for a more precise expression for metabolic disorders [74].

### 4.3. 3D Printing and Advanced Microfabrication

3D printing technologies such as stereolithography (SLA), two-photon polymerization (2PP), and fused deposition modeling (FDM) enable unprecedented control over the geometry of the MN, as well as its porosity and internal structures [75,76]. These techniques enable the rapid prototyping of customizable arrays with hollow channels, microreservoirs, or hybrid designs, which are impossible with traditional etching or molding processes. For example, 3D-printed hollow MNs with integrated ultrasonic atomizers enable on-demand drug atomization, improving drug bioavailability in remote healthcare applications. Advancements in materials include biocompatible resins, e.g., poly(ethylene glycol) diacrylate (PEGDA) to support complex release profiles (sequential multi-drug release for wound healing) [77,78] and additions of 2PP-printed MNs for brain-targeted delivery for crossing the blood–brain barrier with tailored tips for precision neuroscience. High-resolution printing enables patient-specific design and reduces fabrication time (hours) and costs [79,80]. In this context, Pere CPP et al. employed stereolithography-based 3D printing to fabricate polymeric MN patches for transdermal insulin delivery. Regardless of MN geometry, insulin release was completed within 30 min. These findings demonstrate the feasibility of 3D printing as an effective strategy for producing biocompatible microneedle patches with potential for scalable manufacturing [81].

### 4.4. Wearable Patches and Digital Health Integration

Wearable MN patches combine drug delivery with biosensing and Internet of Things (IoT) connectivity to create “closed loop” systems for continuous health management [82]. These platforms detect biomarkers in ISF, such as glucose, electrolytes, or cfDNA, using integrated electrodes or optical sensors, and can automatically initiate dosing based on the detected physiological signals [83,84]. For example, systems based on hydrogels, such as mPatch, have used a set of sensors (CMOS) to monitor the optical concentration of Ca^2+^ ions to provide real-time feedback in metabolic disorders [85,86]. Recent advances include graphene-composite MN patches for painless, non-bleeding monitoring, connected to smartphones for remote data processing and warnings. Integration with AI algorithms can be used to optimize therapy, for example, in patches known as continuous glucose monitoring (CGM), which adjust insulin release to optimize glucose control and improve glycemic control [87,88]. Cloud-based systems help provide telemedicine, enhancing adherence to diabetes and cardiovascular diseases. Miniaturized designs ensure comfort, with battery life > 24 h, though power efficiency and sensor accuracy in dynamic environments are ongoing focuses [89,90].

### 4.5. Personalized and Controlled Release Designs

Progress in polymer chemistry and MN architecture paves the way for customized release kinetics and allows the drugs to have precision medicine and individualized pharmacokinetics [91]. Dissolving MNs with multi-layered tips allows for the sequential release of actives, which is ideal for the combination therapy approach in photoaging or infections [92]. Hydrogel MNs provide extended delivery for days through swelling-controlled diffusion, and hybrid formulations include pulsatile or on-demand delivery pumps [93]. Also, MN patches modulate drug release in response to pH changes at postoperative incision sites, enabling personalized and sustained analgesia beyond conventional invasive pain treatments [94]. Future personalization via AI-driven fabrication promises adaptive therapy, though regulatory standardization is needed [95].

**Table 2:** Innovations in MN Technologies: Features and Applications.

Innovation	Feature	Application	Advantage	Examples	References
Stimuli-responsive MNs	pH-, glucose-, temperature-, H_2_O_2_-sensitive materials	Insulin, targeted cancer therapy	On-demand, closed-loop release	Glucose-responsive insulin MN patches	[60]
Nanoparticle (NP)-loaded MNs	Drug-loaded NPs or liposomes	Vaccines, biologics, gene therapy	Improved stability, targeted delivery	PLGA NPs-loaded MNs	[70]
3D-printed MNs	Customized geometry, multi-layered	Personalized medicine, combination therapy	High precision, rapid prototyping	MN patches for transdermal insulin delivery	[81]
Wearable MN patches	Integrated sensors and electronics	Chronic disease monitoring, digital health	Remote monitoring, automated dosing	Wearable MN patch for monitoring glucose	[87,88]
Hybrid MNs	Combination of dissolving, solid and hydrogel	Multi-drug or sequential release	Optimized pharmacokinetics, patient-tailored therapy	Hybrid dissolving–hydrogel MNs for biphasic release of small molecules such as ibuprofen	[96]

### 4.6. Advances in Smart Microneedle Design: 4D Printing and AI Optimization

Recently, the fabrication of MNs has advanced significantly, driven by 4D printing and bio-inspired designs, to address the longstanding challenge of balancing mechanical strength and biocompatibility. Researchers have developed MN arrays made from dual-sensitive polymers using projection micro-stereolithography, which respond to physiological stimuli, such as moisture, by deploying backward-facing barbs inspired by creatures like parasites and honeybees. This dynamic shape change greatly improves tissue adhesion, reducing the “pop-off” effect and enhancing the utility of MNs for applications like sustained drug release and continuous biosensing [97]. Concurrently, machine learning techniques are revolutionizing MN design by integrating finite element analysis with Gaussian Process Regression to optimize needle geometry and achieve maximum safety margins, ensuring reliable skin penetration without mechanical failure. These scientific and computational innovations mark a shift from static to smart, bioinspired MN architectures with optimized functionality and patient compliance [98].

Collectively, these innovations in smart materials, NP integration, and digital health platforms expand MNs beyond passive delivery devices into multifunctional therapeutic systems. Section 5 translates these technological advances into real-world clinical applications, illustrating how specific MN designs are matched to therapeutic needs across diverse disease areas.

## 5. Therapeutic Applications of Microneedles

**Figure 9:**
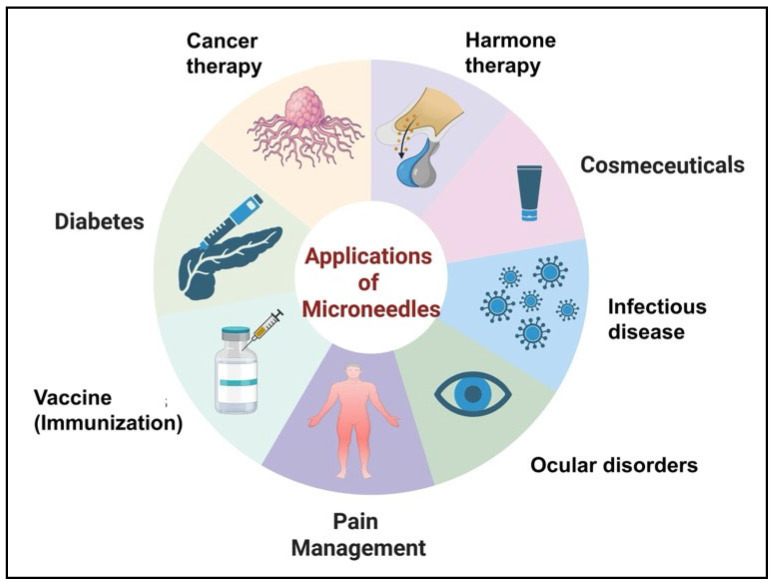
Overview of major applications of MNs in the biomedical and pharmaceutical fields (created in Biorender, Godugu, D. (2025) https://BioRender.com/bidscs4, accessed on 17 December 2025).

MN technologies have expanded beyond the simple transdermal delivery of therapeutics, providing versatile platforms for a broad range of therapeutic applications [99]. Their ability to achieve efficient intradermal delivery with improved patient compliance and bioavailability positions MNs as a competitive replacement for conventional injectable strategies in vaccines, biologics, chronic disease therapeutics, oncology, and cosmetic interventions (Figure 9) [5].

### 5.1. Vaccines and Immunotherapy

Vaccination represents one of the earliest and most extensively studied applications of MNs [100]. Both coated and dissolving MNs have been successfully used for the delivery of influenza, measles, rubella, and hepatitis B vaccines, as well as novel candidates for the administration of the COVID-19 vaccines [101]. MN-mediated delivery targets antigen-presenting cells in the epidermis and dermis, which results in robust humoral and cellular immune responses at lower antigen doses compared to intramuscular injection [102]. This “dose-sparing” effect is critical for maximizing the coverage of costly or supply constrained vaccines [101,103]. Furthermore, MN patches offer a pain-free administration route, which significantly improves patient acceptance, particularly in pediatric populations [104]. Another significant advantage of MN technology is its potential to enhance thermostability. Stabilizing vaccines in a solid-state MN matrix can eliminate the reliance on the cold chain, thereby facilitating distribution to remote or resource-limited areas [105]. Clinical trials have demonstrated the high efficacy of MN influenza vaccines, and research is actively scaling these systems for mass immunization [106]. Beyond prophylaxis, MN technology is also being explored for cancer immunotherapy, delivering agents to specific cutaneous sites to modulate the immune system against malignancies [107].

### 5.2. Diabetes and Peptide Delivery

MN platforms have revolutionized the delivery of insulin and other labile peptide therapeutics. Dissolving and hydrogel-forming MNs provide tunable, extended-release profiles that eliminate the burden of frequent subcutaneous injections [108]. A major advancement in this field is the development of “smart”, glucose-responsive MNs. These closed-loop systems mimic pancreatic function by triggering insulin release only when local glucose levels are elevated, thereby reducing the risk of hypoglycemia [8]. Clinical studies have shown that insulin patches can improve glycemic control and increase patient compliance with therapy. The scope of MNs extends to other peptides, including glucagon-like peptide-1 (GLP-1) analogs and parathyroid hormone fragments, offering a painless and anxiety-free strategy for chronic metabolic and skeletal diseases [47].

### 5.3. Cancer Therapy and Chemotherapy

MN technologies have been adapted for the localized delivery of chemotherapeutics, immunomodulators, and gene therapy vectors, addressing the limitations of systemic administration. Hollow and dissolving MNs enable the controlled delivery of cytotoxic drugs, such as doxorubicin, paclitaxel, and cisplatin-loaded NPs, directly into the tumor microenvironment. This approach enhances intra-tumoral penetration while significantly reducing systemic toxicity [109,110]. MN-based immunotherapy, such as checkpoint inhibitors and vaccine adjuvants, has also shown promise in preclinical models of melanoma and breast cancer by activating local immune responses [111,112]. To further refine therapeutic outcomes, hybrid MN systems combining NPs and stimuli-responsive matrices have been engineered to facilitate spatiotemporal control of drug release [113]. Furthermore, recent advancements have introduced synergistic therapies; hybrid MN systems can combine chemotherapy with photothermal (PTT) or photodynamic therapy (PDT). By incorporating near-infrared responsive agents, these MNs allow for spatiotemporal control of drug release and thermal ablation, maximizing anti-cancer efficacy while minimizing off-target effects [114].

### 5.4. Hormonal and Contraceptive Delivery

MN patches offer a discreet and convenient alternative for hormonal therapies, including contraception, hormone replacement, and fertility treatments. Dissolving MNs have been engineered to release levonorgestrel, estradiol, and progesterone with sustained release kinetics suitable for weekly or monthly dosing [115]. Wei et al. developed a bubble-assisted, rapidly separable microneedle patch enabling manual skin insertion and sustained transdermal delivery of levonorgestrel. The microneedles detached under low shear stress within seconds of application and achieved prolonged hormone release, with ~95% cumulative absorption over 45 days and approximately 70% bioavailability in vivo [116]. By removing the need for trained healthcare personnel to administer injectable contraceptives, MNs have the potential to significantly increase access to family planning, particularly in low-resource settings [117].

### 5.5. Cosmeceuticals and Dermatology

The cosmetic and dermatology sectors have embraced MNs for skin rejuvenation, pigmentation correction, and transdermal delivery of growth factors, peptides, and vitamins. MNs create physical micro-channels that bypass the stratum corneum, significantly increasing the penetration of molecules such as hyaluronic acid, retinoids, peptides, and antioxidants, which otherwise exhibit poor dermal absorption [118]. Dissolving MNs deliver these compounds painlessly while the mechanical action of the needles simultaneously triggers a natural wound-healing cascade, inducing collagen and elastin production [119]. Consequently, MN-based cosmeceuticals have gained popularity as minimally invasive, “office-free” alternatives to clinical procedures, offering reduced infection risks and shorter recovery times compared to traditional microneedling rollers [27].

### 5.6. Infectious Disease Therapeutics

Beyond vaccination, MNs are increasingly explored for the treatment of viral and bacterial infections. Dissolving MNs can deliver antiviral peptides, nucleic acids, or antibiotics directly to the site of infection in the dermis, enhancing local efficacy while mitigating systemic side effects. Studies targeting herpes simplex virus, human papillomavirus (HPV), and bacterial skin infections have shown that MNs achieve superior local drug concentrations compared to topical creams [120,121]. Furthermore, long-acting antiretroviral therapy can be effectively delivered via intradermal administration with dissolving or implantable MN patches, overcoming adherence challenges associated with oral and injectable dosing. Incorporation of etravirine and rilpivirine nanosuspensions into dissolving arrays has demonstrated efficient transdermal deposition of drug nanocrystals and markedly enhanced systemic and lymphatic exposure in vivo [122,123]. Subsequent designs co-loading cabotegravir and rilpivirine have yielded sustained plasma concentrations for several weeks following a single application, with repeat dosing maintaining prolonged therapeutic levels [124]. More recent systems embedding bictegravir and tenofovir alafenamide further confirmed that MN-mediated intradermal delivery can achieve sustained systemic concentrations of integrase inhibitors while enabling rapid conversion of prodrugs, highlighting the platform’s strong potential for long-acting treatment and pre-exposure prophylaxis [125].

### 5.7. Ocular Therapeutics

A rapidly emerging application of MNs is the treatment of ocular diseases, particularly those affecting the posterior segment of the eye, such as age-related macular degeneration (AMD) and diabetic retinopathy. Conventional eye drops suffer from poor bioavailability due to corneal barriers, while intravitreal injections carry risks of endophthalmitis and retinal detachment. MNs designed for corneal or intrascleral application offer a minimally invasive route to deliver therapeutics directly to ocular tissues [126]. Specialized MNs have been developed to deliver anti-VEGF agents and corticosteroids, demonstrating sustained release and therapeutic concentrations in the retina and choroid with reduced invasiveness compared to traditional needles [14].

### 5.8. Pain Management and Local Anesthesia

MNs provide an effective solution for rapid and painless local anesthesia. Conventional hypodermic needles used for anesthetic delivery cause pain and anxiety, often requiring topical pre-treatment. MN arrays loaded with anesthetics like lidocaine or prilocaine offer a “press-and-patch” solution that ensures the rapid onset of anesthesia by delivering the drug directly to dermal nociceptors [127,128]. This application is particularly valuable for pediatric procedures, minor dermatological surgeries, and management of neuropathic pain conditions, offering a user-friendly alternative to painful injections [25].

Despite compelling preclinical and clinical outcomes across therapeutic areas, widespread clinical adoption of MN technologies remains constrained by manufacturing complexity, regulatory classification, and market acceptance. These translational challenges are discussed in detail in Section 6.

## 6. Commercial Challenges, Regulatory Pathways, and Case Studies

**Figure 10:**
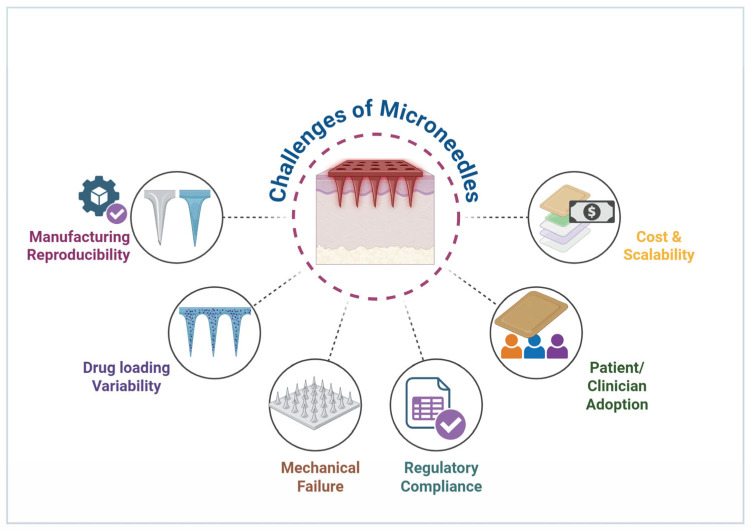
Key challenges in MN technology development (created in Biorender, Godugu, D. (2025) https://BioRender.com/ni6s0wt, accessed on 25 November 2025).

Despite the promising therapeutic potential of MN technologies, the translation from laboratory prototypes to commercially viable products is hindered by complex barriers in manufacturing, regulation, and reimbursement. While technical feasibility has been established, the “Valley of Death” for MNs lies in achieving consistent bioequivalence at an industrial scale and navigating the bifurcated regulatory landscape for drug-device combination products [129]. Addressing these barriers is critical for widespread adoption in clinical and consumer settings (Figure 10).

### 6.1. Manufacturing and Scalability Challenges

A key obstacle to scaling up MN production is consistently achieving uniform quality and performance across large batches. Ensuring that MN arrays maintain precise dimensions, consistent sharpness, and intact tips throughout manufacturing is critical, as even minor dimensional deviations can compromise insertion efficiency and therapeutic performance. For dissolving or coated MNs, this challenge is compounded by the need to precisely control polymer composition, coating thickness, and drug loading to ensure dose uniformity and reproducible release profiles; minor variations in coating thickness can lead to pharmacokinetic (PK) variability, a regulatory dealbreaker. Transitioning from laboratory-scale fabrication to industrial production presents significant challenges, especially with complex MN designs and drug-device combination products [130,131]. Furthermore, maintaining the mechanical robustness and drug stability of MNs, particularly those containing delicate biologic agents, is vital for prolonged shelf life. Despite promising clinical outcomes, large-scale manufacturing remains a significant bottleneck. Additional concerns include the need for aseptic processing to produce vaccine-loaded MNs, which is more intricate and expensive than conventional sterilization methods [48]. However, significant progress was made in 2025, with Vaxxas securing a Therapeutic Goods Administration (TGA) license for its robotic aseptic manufacturing line, marking a pivotal shift from pilot to commercial-scale capability [132]. Similarly, Micron Biomedical successfully demonstrated the scalability of its dissolving microneedle platform in a landmark 2024 Phase 1/2 trial for measles-rubella vaccination in infants (NCT04394689). Moreover, ensuring consistent application force through standardized applicators, like the spring-loaded devices used by Vaxxas, is becoming a regulatory expectation to reduce variability in insertion depth and minimize user errors [133,134]. While advanced fabrication approaches such as 3D printing enable unprecedented design flexibility and patient-specific customization, their current high cost, limited material approval, and lower throughput restrict large-scale clinical deployment. In contrast, micro molding and roll-to-roll manufacturing offer superior scalability and regulatory familiarity, explaining why most clinically advanced dissolving MN products rely on these methods. Bridging this gap between innovation and manufacturability remains a central translational challenge for next-generation MN systems.

### 6.2. Regulatory Approval Pathways

The approval of MN-based technologies varies depending on their design, function, and degree of drug involvement [135]. In the United States, MN systems that do not contain a drug at the point of sale, including hollow microneedles used to deliver separately approved formulations, are regulated as Class II medical devices under the FDA’s Center for Devices and Radiological Health (CDRH). These products may reach the market via the 510(k) pathway by demonstrating substantial equivalence to predicate hypodermic needles, with regulatory focus on mechanical performance and safety rather than drug efficacy. However, labeling is typically limited to intradermal delivery of substances already approved for that route, restricting product-specific marketing and often relegating clinical use to off-label practice [136]. Dissolving and drug-coated MN patches are regulated as combination products, as the drug and device form an inseparable single entity. Jurisdiction is determined by PMOA, with therapeutic patches overseen by either CDER or CBER. Importantly, the FDA considers MN patches to be novel dosage forms; incorporation of an already approved drug does not constitute a simple reformulation. Manufacturers must demonstrate bioequivalence to the reference product, and deviations in pharmacokinetic profiles may necessitate a full 505(b)(2) New Drug Application. Combination products must also comply simultaneously with device quality system regulations and pharmaceutical cGMPs, substantially increasing manufacturing and regulatory complexity [136,137,138].

In Europe, the Medical Device Regulation (MDR, EU 2017/745) has further tightened oversight. Under MDR Rule 14, devices incorporating an integral medicinal substance are classified as Class III. At the same time, products whose principal action is pharmacological, immunological, or metabolic are regulated as medicinal products under Directive 2001/83/EC. As a result, dissolving MNs delivering drugs or vaccines generally require a full Marketing Authorization rather than CE marking. Additionally, MDR Article 117 mandates consultation with a medicines authority for integral drug-device combinations regulated as devices, adding time and cost to conformity assessment [130].

Overall, the lack of global regulatory harmonization means similar MN technologies may be classified differently across jurisdictions, reinforcing the importance of early regulatory strategy and agency engagement during product development.

### 6.3. Case Studies of Marketed and Trial-Stage MN Products

Several MN platforms have progressed from laboratory research to advanced clinical trials and limited market entry, underscoring both the technological maturity and remaining translational challenges of this field (Table 3). These case studies highlight key innovations, clinical outcomes, and development barriers such as mechanical reliability, drug stability, manufacturing scalability, regulatory classification, and cost management [139].

Sanofi Pasteur, with Becton Dickinson, marketed as Intanza^®^ in Europe and Fluzone^®^ Intradermal in the United States, is delivered via the BD Soluvia™ microinjection system [140]. However, the Intanza^®^ product was discontinued due to limited manufacturing scalability, lack of economies of scale, and the rapid market shift to quadrivalent influenza vaccines, underscoring that clinical efficacy alone is insufficient for sustained commercialization [141]. In contrast, Zosano Pharma’s Qtrypta™, a zolmitriptan-coated microneedle patch for migraine treatment, encountered regulatory setbacks due to manufacturing-driven pharmacokinetic variability rather than a lack of therapeutic efficacy. FDA concerns regarding inconsistent systemic exposure underscored the critical importance of dose uniformity and bioequivalence in microneedle-based combination products. Although the FDA requested additional bioequivalence data rather than rejecting the product, financial constraints ultimately led to the discontinuation of the program [142].

An alternative strategy is exemplified by NanoPass Technologies’ MicronJet™, a hollow microneedle device cleared via the FDA 510(k) pathway as a general-purpose injection tool. By decoupling the device from the drug, NanoPass achieved rapid market entry, albeit with limitations on product-specific therapeutic claims. Emerging platforms, including Vaxxas’ high-density microarray patch and Micron Biomedical’s dissolving microneedle system, have integrated these lessons by prioritizing manufacturing control (robotic aseptic manufacturing) and regulatory alignment [143,144].

**Table 3:** Summary of representative MN products and their clinical development status.

MN Type	Product/Platform	Company	Therapeutic Area	Status/Outcome	Reference
Solid/coated	High-density MNs patch	Vaxxas	Influenza (H7N9)	Phase I (H7N9); TGA manufacturing license secured.	[132], NCT06417853
Dissolving	Dissolvable MNs patch	Micron Biomedical	Vaccines (Measles-Rubella, Rotavirus)	Phase 1/2 (MR) published 2024. Phase I (Rotavirus) launched June 2025 with the CDC.	[145,146]
Solid/coated	emxRNA™ Patch	Kindeva/Emervax	mRNA Vaccines	Preclinical. Partnership in announced January 2025. Clinical trials anticipated 2026.	[147,148]
Solid/coated	Qtrypta (M207)	Emergex Vaccines (ex-Zosano)	Infectious Diseases	Phase I. Acquired Zosano assets (2022) post-bankruptcy. Qtrypta migraine program discontinued.	[149,150]
Solid Coated Patch	Abaloparatide-sMTS	Radius Health/Kindeva	Osteoporosis	Discontinued (2022). Phase 3 wearABLe trial failed the non-inferiority endpoint vs. injectable.	[151], NCT04064411
Hollow Microneedle (MEMS)	MicronJet™	NanoPass Technologies	Aesthetics, Vaccines	Marketed (510 k Cleared)	[103]

### 6.4. Market Adoption and User Acceptance

Even after receiving regulatory approval, the success of commercializing these products hinges on their acceptance by patients, healthcare providers, and institutions [103]. Patient preference is a significant factor, as the painless and self-administered nature of MN patches makes them particularly appealing for chronic diseases and vaccinations in children [152]. However, for these products to gain acceptance, healthcare providers need to provide training, demonstrate proven efficacy, and offer reassurance about safety. Additionally, economic factors play a crucial role. The cost-effectiveness of MN products, along with favorable reimbursement strategies and pricing models, will ultimately influence their adoption, especially in resource-limited regions [153]. Although the high manufacturing costs associated with early MN technologies may hinder widespread acceptance, ongoing research aims to develop a more affordable and efficient manufacturing process.

## 7. Future Perspectives

MN technologies have evolved from experimental concepts to highly promising platforms for transdermal and intradermal drug delivery with expanding therapeutic applications. Advances in smart and responsive materials enable MNs to respond to physiological stimuli, such as glucose, temperature, and pH, enabling precise, on-demand drug release. Integration with digital health technologies further enhances their function by supporting real-time monitoring and personalized treatment adjustments [9,153,154]. Hybrid MN systems that combine drug delivery with diagnostic capabilities are emerging, offering more comprehensive patient care. MNs also hold great potential for global health by facilitating needle-free, thermostable vaccine delivery suitable for resource-limited settings [155]. However, challenges remain in scalable manufacturing, regulatory standardization, biocompatibility, and patient education [139,156]. Ongoing research into biodegradable polymers, stimuli-responsive compounds, and collaborative efforts across academia, industry, and regulators will be crucial to overcoming these hurdles and accelerating clinical translation.

## 8. Conclusions

MN technologies have been revolutionary in drug delivery systems as they are minimally invasive, support positive patient experiences, and offer potential for customization to individual therapeutic needs. Beyond improving adherence, MN systems provide remarkable clinical versatility, enabling the transdermal delivery of a wide range of therapeutics, including vaccines, biologics, small molecules, and combination therapies. Their structural flexibility allows integration with advanced responsive materials and wearable biosensors, facilitating “on-demand” drug release and real-time physiological monitoring. On a global scale, MN technologies hold promise for portable, self-administered healthcare solutions, from chronic disease management to needle-free vaccination programs, particularly in resource-limited settings. Self-administered MN vaccines can reduce dosing frequency while improving therapeutic outcomes and adherence. However, the successful translation of MN systems into widespread clinical use will depend on overcoming key challenges, including scalable, reproducible manufacturing; long-term biocompatibility; regulatory harmonization across regions; and effective patient education. Addressing these hurdles will be critical to fully realizing the therapeutic and societal potential of MN technologies.

## Data Availability

No new data were created or analyzed in this study.
